# Rhizosphere Protists Change Metabolite Profiles in *Zea mays*

**DOI:** 10.3389/fmicb.2018.00857

**Published:** 2018-05-03

**Authors:** Anke Kuppardt, Thomas Fester, Claus Härtig, Antonis Chatzinotas

**Affiliations:** ^1^Department of Environmental Microbiology, Helmholtz Centre for Environmental Research – UFZ, Leipzig, Germany; ^2^German Centre for Integrative Biodiversity Research (iDiv) Halle-Jena-Leipzig, Leipzig, Germany

**Keywords:** predator-prey interactions, trophic interactions, metabolites, microcosms, rhizosphere microorganisms

## Abstract

Plant growth and productivity depend on the interactions of the plant with the associated rhizosphere microbes. Rhizosphere protists play a significant role in this respect: considerable efforts have been made in the past to reveal the impact of protist-bacteria interactions on the remobilization of essential nutrients for plant uptake, or the grazing induced changes on plant-growth promoting bacteria and the root-architecture. However, the metabolic responses of plants to the presence of protists or to protist-bacteria interactions in the rhizosphere have not yet been analyzed. Here we studied in controlled laboratory experiments the impact of bacterivorous protists in the rhizosphere on maize plant growth parameters and the bacterial community composition. Beyond that we investigated the induction of plant biochemical responses by separately analyzing above- and below-ground metabolite profiles of maize plants incubated either with a soil bacterial inoculum or with a mixture of soil bacteria and bacterivorous protists. Significantly distinct leaf and root metabolite profiles were obtained from plants which grew in the presence of protists. These profiles showed decreased levels of a considerable number of metabolites typical for the plant stress reaction, such as polyols, a number of carbohydrates and metabolites connected to phenolic metabolism. We assume that this decrease in plant stress is connected to the grazing induced shifts in rhizosphere bacterial communities as shown by distinct T-RFLP community profiles. Protist grazing had a clear effect on the overall bacterial community composition, richness and evenness in our microcosms. Given the competition of plant resource allocation to either defense or growth, we propose that a reduction in plant stress levels caused directly or indirectly by protists may be an additional reason for corresponding positive effects on plant growth.

## Introduction

The rhizosphere is a hotspot of microbial interactions (Bakker et al., 2013). It is densely populated with members from all domains of life and characterized by myriads of interactions (Bonkowski et al., 2009; Raaijmakers et al., 2009; Jacoby et al., 2017). Plant roots are key drivers of this habitat by releasing low and high-molecular weight carbon compounds into the soil in order to lubricate their root tips or by losing exudates through leaky root tips (Farrar et al., 2003; Hartmann et al., 2009). This plant-derived carbon lifts the C-limitation in soil leading to rapid bacterial growth, higher activity and microbial community shifts (Paterson, 2003; Jones et al., 2009; Steinauer et al., 2016), which in turn mobilizes nutrients from soil organic matter, in particular nitrogen. Nitrogen together with other nutrients locked up in the microbial biomass like bacteria or fungi is released by protistan grazing and serves again as nutrition for bacterial and plant growth (Bonkowski, 2004; Ekelund et al., 2009; Bonkowski and Clarholm, 2012; Koller et al., 2013b). In addition to the enhanced nutrient availability driven by the microbial loop, protist may also influence root architecture, exemplified by a strong growth stimulation of lateral roots in *Lepidium sativum* L., *Oryza sativa* L. and *Arabidopsis thaliana* (Bonkowski and Brandt, 2002; Kreuzer et al., 2006; Krome et al., 2010). Enhanced root branching in turn fosters growth and activity of soil bacteria by the increased release of carbon rich photosynthates.

Selective protistan grazing directly or indirectly shifts the microbial community composition in soil (Rosenberg et al., 2009). Bonkowski and Brandt (2002) provided some evidence that grazing may in particular result in an increase of the abundance and activity of plant growth promoting rhizobacteria (PGPR). Various mechanisms for the effects of PGPR on plants have been described like antagonism to fungal pathogens, enhancing nutrient availability like phosphate (Hassan et al., 2010) or iron (Sayyed et al., 2013) and the release of bacterial volatiles as inducer of systemic resistance (Ping and Boland, 2004). The production of 1-aminocyclopropane-1-carboxylate (ACC) deaminase (Ma et al., 2003) reduces ethylene levels and thus facilitates plant growth following an environmental stress (Friesen et al., 2011). Another important function of PGPR is the synthesis of the plant hormone indole-acetic acid (IAA) (Dobbelaere et al., 2001; Patten and Glick, 2002), which is the master regulator for the initiation of lateral root primordia and root elongation (Aloni et al., 2006). This influence of PGPR on root architecture is similar to the enhanced formation of lateral roots in the presence of protists due to the proportional increase of IAA-producing bacteria by grazing (Bonkowski and Brandt, 2002).

The impact of protist-bacteria interactions in the rhizosphere on plant productivity and plant architecture has been described repeatedly in *Lepidium sativum* L., *Plantago lanceolata* L., *Oryza sativa* L., and *Arabidopsis thaliana* (Bonkowski and Brandt, 2002; Kreuzer et al., 2006; Krome et al., 2010; Koller et al., 2013b). Research on the interactions between rhizosphere microbes and plants has focused primarily on plant diseases, defense mechanisms and the influence of PGPR so far. Reports include for example changes in root gene expression in response to the presence of pathogenic bacteria (Chen et al., 2014) and PGPR (Camilios-Neto et al., 2014; Plucani do Amaral et al., 2014) as well as changes in plant metabolites after incubation with PGPR and mycorrhiza (Singh et al., 2002; Dhawi et al., 2015; Gupta et al., 2017). However, the bottom-up effects of protist-bacteria interactions in the rhizosphere on the plant metabolic state have not been described so far. Here, we extend metabolite profiling (Fester et al., 2011, 2014) to the ecological research field of rhizosphere microbes and plant interactions. In addition to the established method of nutrient analysis the method of metabolite profiling allows a holistic and sensitive image of the state of a plant organism.

In an attempt to gain a first insight into the plant metabolic responses to protist-bacteria interactions in the rhizosphere, we exposed plants to microbial communities differing in their trophic levels and comparatively analyzed the resulting metabolite profiles in leaves and roots. For this study we used a model laboratory system with *Zea mays* L., growing in the presence of a natural bacterial soil community either with or without selected bacterivorous protists. These protists represent commonly detected free-living protists in soils and cover different feeding modes such as flagellum-mediated filter feeding (*Cercomonas longicauda*, Pedersen et al., 2008; Bass et al., 2009*)*, cilium-mediated filter feeding (*Tetrahymena pyriformis*, Fenchel, 1987; Parry, 2004) and surface gliding and feeding (*Acanthamoeba polyphaga*, Clarholm, 1981; Weekers et al., 1993*)*. We hypothesized that the presence of protists does not only affect the overall bacterial community structure via trophic interactions and the overall plant performance, but also results in distinct metabolites in different compartments of the model plant.

## Materials and Methods

### Experimental Setup

The experimental setup was based on the procedure described by Rosenberg et al. (2009). Twenty magenta vessels (Magenta.GA-7; Magenta LLC; Lockport, IL, United States) were filled with 200 g dry weight of sand (Spielsand, Hagebaumarkt Leipzig) and 0.5 g of milled hay (Winston Bergwiesenheu; Rossmann Leipzig). The magenta vessels were autoclaved three times with pauses of 2 days in between. Sterility of the sand/hay mix was checked by plating on nutrient broth agar (NB; Merck, Darmstadt, Germany).

The vessels were inoculated with a protist-free natural bacterial community that was gained by filtration of a soil sample derived from a flowerbed (campus of the Helmholtz Centre for Environmental Research – UFZ, Leipzig). Ten grams of this sample were suspended with 50 ml of autoclaved tap water and shaken for 1 h. The soil slurry was subsequently filtered through an 8.0 μm (Whatman GmbH; Dassel, Germany), a 3.0 and a 1.2 μm filter (Merck Millipore; Burlington, MA, United States), respectively. To check for protist contaminations, the filtrate was concentrated on a 0.2 μm filter (Merck Millipore; Burlington, MA, United States) and this filter was used for DNA extraction as described below and subsequent 18S rRNA gene PCR. A second filter was used for cell counting with a microscope (Axioskop 20; Carl Zeiss, Göttingen, Germany) after DAPI staining for 10 min in the dark (2 μg/ml; Invitrogen/ Life Technologies; United States). Stained filters were immersed with 20 μl Citifluor (Citifluor Ltd.; London, United Kingdom) and 400 squares were counted. For soil inoculation 7.5 ml (i.e., 9 × 10^6^ cells) of the extracted bacterial community were added to each culture vessel and thoroughly mixed with the sand/hay mixture. After 3 days half of the vessels (*n* = 10) were used for a subsequent inoculation with three different bacterivorous protists.

Axenic (i.e., bacterium free) cultures of a ciliate (*Tetrahymena pyriformis)*, an amoebae (*Acanthamoeba polyphaga)* and a flagellate (*Cercomonas longicauda)* were grown as described by Saleem et al. (2013) and concentrated by centrifugation at 26 g for 15 min and subsequently washed in autoclaved tap water. Ten culture vessels received 1.5 ml of a mixed protist inoculum, while the other ten vessels received 1.5 ml of tap water. Cell numbers were established via direct counting in a cell chamber. Protists were fixed with Lugol solution and at least 3 × 20 squares were counted under the microscope. Cell abundances in the mixed protist inoculum were as follows: *Tetrahymena pyriformis* 1.8 × 10^5^/ml, *Acanthamoeba polyphaga* 3.6 × 10^4^/ml and *Cercomonas longicauda* 7.5 × 10^4^/ml. These three organisms were chosen as they cover different feeding modes such as flagellum-mediated filter feeding (the flagellate), cilium-mediated filter feeding (the ciliate) and surface gliding and feeding (the amoeba). The used inoculation levels were in the range of measured abundances of protists in sandy soil with low organic C content (Verhoeven, 2002).

Five of the ten inoculated soil vessels received a second 0.5 ml mixed inoculum after another 8 days with following cell abundances: *Tetrahymena pyriformis* 5.0 × 10^4^/ml, *Acanthamoeba polyphaga* 2.2 × 10^5^/ml, and *Cercomonas longicauda* 2.7 × 10^4^/ml, while the other fifteen vessels received 0.5 ml of tap water.

*Zea mays* L. cv. Rivaldo seeds were sterilized prior to cultivation using 15% hydrogen peroxide for 5 min. The seeds were washed three times in autoclaved tap water and afterwards cultivated in sterile watered tissue in darkness. Five days old seedlings were used for transplantation to the soil culture vessels 2 days after the first inoculation with protists. From this time on the vessels were kept under unsterile conditions as the lids contained an opening for the plants and the addition of water. Plants were watered every second day with 2 ml of autoclaved tap water.

### Sampling

Samples were taken after 14 days of plant growth after planting in vessels. Plant growth was determined by measuring the shoot diameter as well as the shoot length, and by counting the number of leaves. From each plant the third leaf and the complete root was sampled, immediately frozen in liquid nitrogen and stored for metabolite profiling at -80°C. The sand was divided into three fractions. Soil that remained in the vessel after careful plant removal was thoroughly mixed and defined as bulk soil (bs). The plant with adhering sand was transferred to a sterile beaker and vigorously shaken, resulting in a soil fraction termed rhizosphere soil (rs). Still remaining sand was washed from the root with autoclaved tap water and called rhizosphere soil II (rII). All soil fractions were stored at -20°C for further DNA extraction.

### DNA Extraction, PCR and 16S rRNA Gene T-RFLP Analysis

Genomic DNA was extracted using the NucleoSpin^®^Soil-Kit (Macherey-Nagel; Düren, Germany). Soil samples with a weight of 450–500 mg were used for extraction according to the manufactures instructions with buffer SL1 and enhancer SX. DNA was eluted with 80 μl SE buffer. For the analysis of the terminal restriction fragment length polymorphisms (T-RFLP) we used the 16S rRNA specific primers UniBac27f (Lane, 1991) (6′-FAM labeled) and Univ519r (Lane et al., 1985). PCR was performed in a thermocycler using twofold concentrated PCR Master Mix (Qiagen, Hilden, Germany) and 5 pmol of each primer. PCR conditions consisted of initial denaturation for 4 min at 94°C and 30 cycles of denaturation for 30 s at 94°C, annealing for 45 s at 56°C and elongation for 30 s at 72°C. Final elongation lasted 10 min at 72°C.

The labeled PCR products were purified using SureClean (Bioline; Luckenwalde, Germany) and their quantity was determined by gel quantification. Aliquots of 20 ng were digested over night at 37°C with two units of MspI, HhaI, and AluI (NEB); respectively (Giebler et al., 2013). The total volume of 10 μl of the digestion was precipitated by adding 1 μl 3 M Na-acetate (pH 5.5) and 25 μl ethanol. After centrifugation the pellet was washed with 300 μl 70% ethanol followed by another centrifugation step. The dried pellet was dissolved in 20 μl HIDI mixed with 0.3 μl size standard ROX500 (Applied Biosystems). Fluorescently labeled terminal restriction fragments (T-RFs) were size separated on an ABI 3130 genetic analyzer (Applied Biosystems) and resulting electropherograms were analyzed using the GeneMapper software (Applied Biosystems).

Terminal restriction fragment length polymorphisms data including T-RF sizes between 50 and 500 bp were normalized and standardized by an algorithm that identifies true peaks as those whose area is greater than the standard deviation calculated over all peaks (Abdo et al., 2006) and bins peaks across all samples using cut-off value of five times standard deviation. After normalization, the relative abundance of each T-RF was calculated as the percentage of the combined peak area of each sample.

The 18S rRNA gene PCR performed to check for protist contamination in the bacterial filtrate was done as described in Glaser et al. (2015); DNA from the three protists served as positive controls.

### Metabolite Profiling

Metabolite profiling was based on the procedure described in Sanchez et al. (2008). In short, the frozen material was homogenized in a Retsch ball mill (MM301, Retsch GmbH, Germany) for 3 min at 30 s^-1^ and resuspended in 300 μl methanol at -20°C. After the addition of 30 μl ribitol (0.2 mg/ml dissolved in methanol), 30 μl non-adecanoic acid methylester (2 mg/ml in chloroform) and 30 μl isoascorbic acid (0.5 mg/ml in water), samples were incubated in a shaker for 15 min at 70°C. Subsequently 200 μl chloroform was added, samples were shaken for 5 min at 37°C, mixed with 400 μl of water and vortexed. Phase separation was achieved by centrifugation (5 min, 14 000 rpm). Two 10 μl aliquots from the upper phase were finally dried in vacuum over night at room temperature. The dried material was stored at -80°C and derivatized as described in Desbrosses et al. (2005): The samples were suspended in 80 μl methoxamin hydrochloride (20 mg/ml in pyridine), incubated for 90 min at 30°C; subsequently 80 μl of *N*-methyl-*N*-(trimethylsilyl)-trifluoroacetamide was added and samples were incubated for 30 min at 37°C. Finally 16 μl of a standard mix containing C_10_, C_12_, C_15_, C_18_, C_19_, C_22_, C_28_, C_32_, C_36_ n-alkanes at 0.22 mg/ml was added. Gas chromatography was done using an Agilent GC 6890 equipped with a Rtx-5Sil MS capillary column (30 m × 0.25 mm inner diameter, 0.25 μm film thickness and 5 m integrated guard column; Restek GmbH, Bad Homburg vor der Höhe, Germany) and an MSD 5973. From each sample 1 μl was injected in splitless mode with a 2 min pulse at 110 psi at a temperature of 230°C. Helium was used as carrier gas with constant flow at 1 ml/min. The temperature program was 1 min at 70°C, 1°C/min to 76°C and finally 6°C/min to 350°C, held for 1 min. The transfer line to the mass spectrometer was set to 250°C. Gas chromatography-mass spectrometry data were subjected to baseline correction using MetAlign (Tikunov, 2005); chromatographic deconvolution and quantification of compounds was done using TagFinder (Luedemann et al., 2008). Using this latter program, a retention time index (RI) was calculated from the added *n*-alkanes. Metabolites were identified by comparison of RI-values and fragment masses to the Golm metabolome database (Kopka et al., 2005; Schauer et al., 2005) using the programs TagFinder and AMDIS (NIST).

### Statistical Analyses

Statistical analysis was performed using R version 3.3.0 (R Development Core Team, 2015). Metabolite data were normalized using sample weights and the internal standard ribitol and subsequently logarithmized. Metabolite ratios were calculated by dividing metabolite levels from leaves by metabolite levels from roots, because metabolite ratios comparing sink with source organs can reflect growth parameters of plants (Fester et al., 2013, 2014). In all cases only data from the same plant individuals were used for calculating such ratios. Multivariate ordination analysis of metabolite levels and ratios was performed using partial least square-discriminant analysis (PLS-DA) (Næs and Mevik, 2001; Wold et al., 2001). PLS-DA was performed using the function plsda() from the R-package ‘caret’ (Kuhn et al., 2012). Significant metabolites were plotted to PLS-DAs using the function envfit() from the R-package ‘vegan’ (Oksanen et al., 2012). This function assessed significance of correlations by a correlation test using Monte Carlo permutations (*N* = 999) of the fitted vectors (Hollander and Wolfe, 1973). The goodness of fit statistics used was squared correlation coefficient (Pearson’s product moment correlation coefficient). Significance of the separation of treatment groups in multivariate ordination analysis was tested by permutational multivariate analysis of variance (PERMANOVA, 999 permutations) using the function adonis() from the R-package ‘vegan’. Significance of differences in individual levels or ratios of metabolites when comparing samples with and without added protists was assessed using a two-sided student *t*-test in Microsoft Excel. All metabolites with significant fold changes are listed in **Table 2**. All metabolites that contain either P or N and were shown to be linked to nutrition status of plants before (Fester et al., 2013, 2014) are listed in **Table 3**.

To visualize the dissimilarities in the overall community composition (i.e., the T-RFLP profiles) between the samples non-metric multidimensional scaling (nMDS) plots were calculated based on the Bray-Curtis dissimilarity index (Bray and Curtis, 1957). This index had already been described as suitable for T-RFLP data because it ignores “joint absence” and gives in nMDS plots the best results for T-RFLP data (Culman et al., 2008). The calculation of richness, evenness and Shannon index of T-RFLP data was done using the diversity indices function in PAST Version 2.06. To reveal the impact of protists and soil fractions on the T-RFLP profiles we applied PERMANOVA (999 permutations) with adonis function in R (Anderson, 2001). We used one way analysis of variance (ANOVA) in R to analyze the influence on richness, evenness and Shannon index as well as the impact of protists on plant growth parameters. Before running ANOVA we checked the distribution of data by Shapiro–Wilk normality test and log transformed data if necessary.

## Results

### Plant Growth

**FIGURE 1:**
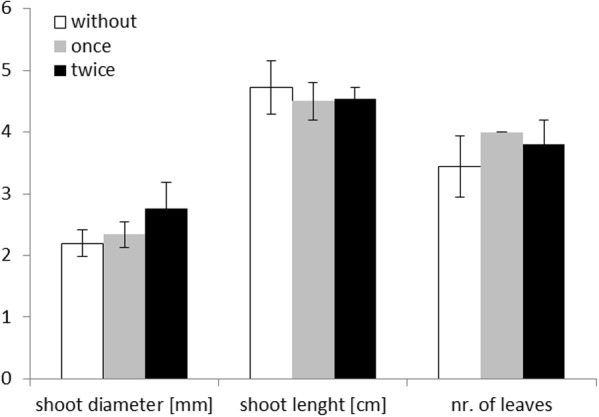
Maize plant measurements on day of harvest (day 14) for the three different treatments: without addition of protists (white; *n* = 10), singular addition (once – gray; *n* = 5) and repeated addition of protists (twice – black; *n* = 5). Mean values are shown with standard deviation as error bars. Shoot diameter is given in mm, shoot length in cm, and number of leaves as numbers.

None of the plant growth parameters did differ significantly between treatments without protists, with a single or with a twofold addition of protists (**Figure 1**). When comparing all treatments with protists and these without protists, a slight increase (*p* = 0.049, *F* = 4.49, degree of freedom: 1,18, one way ANOVA) in mean shoot diameter of *Zea mays* L. grown in the presence of protists was observed on the day of harvest (14 days after planting). A significant influence of protists was measured for the mean number of leaves (*p* = 0.034, *F* = 5.35, degree of freedom: 1,18, one way ANOVA). In the presence of protists most plants (nine out of 10 plants) developed four leaves while this was the case for only five out of 10 plants growing without protists.

### Bacterial Community Structure

**FIGURE 2:**
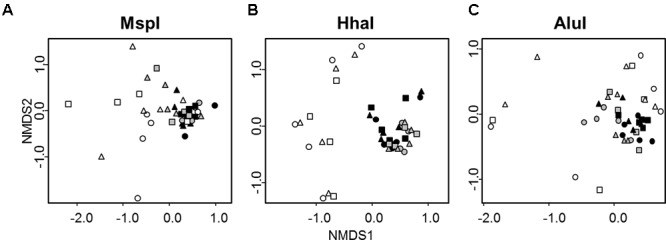
nMDS plots of T-RFLP profiles of bacterial communities obtained after digestion with the three different restriction enzymes MspI **(A)**, HhaI **(B)**, and AluI **(C)** from three soil fractions (bulk soil – circles, rhizosphere soil – triangles – adhering sand to the maize root which was gained by shaking the root, rhizosphere II – squares – remaining sand which was washed from the root). Samples not treated with protists are represented in white (*n* = 10), samples treated with protists once in gray (*n* = 5), and samples treated twice with protists in black (*n* = 5). Stress value = 0.14.

Differences in the overall bacterial community composition of each of the three soil fractions (bulk soil – bs, rhizosphere soil – rs, rhizosphere soil II – rsII) were assessed by T-RFLP of 16S rRNA genes with three different restriction enzymes. T-RFLP profiles of bacterial communities not exposed to protists were well separated in nMDS plots from profiles of communities interacting with protists irrespective of the used restriction enzyme (**Figure 2**). No separation was observed between samples receiving singular or twofold protist inoculations. PERMANOVA analysis revealed that bacterial community composition was mainly explained by the presence of protists (**Table 1**); protists had a significant positive influence on diversity (Shannon Index), richness and evenness for the two enzymes AluI and HhaI. In the MspI treatment protists reduced significantly diversity and richness, while evenness was unchanged. In contrast, the soil fractions as sampled in our study did not show any significant influence on any community parameter (**Table 1**).

**Table 1:** Influence of protist addition and soil fraction on the bacterial community composition (estimated by PERMANOVA) for three different restriction enzymes.

	MspI		HhaI		AluI	
	Protists (d*f* = 1,42)	Soil fraction (d*f* = 2,42)	Protists (d*f* = 1,42)	Soil fraction (d*f* = 2,42)	Protists (d*f* = 1,42)	Soil fraction (d*f* = 2,42)
Community composition	5.40^∗∗∗^	1.74	11.74^∗∗∗^	1.57	4.45^∗∗∗^	1.45
Richness	2.61^∗^	0.35	11.31^∗∗∗^	0.02	17.59^∗∗^	2.31
Evenness	2.34	3.23	15.87^∗∗∗^	0.90	10.18^∗∗^	0.23
Shannon index	3.68^∗^	0.69	24.95^∗∗∗^	0.43	24.63^∗∗∗^	0.31

*The impact on different diversity indices was analyzed using one way ANOVA. Vessel sand was divided into three fractions: bulk soil (bs), rhizosphere soil (rs, adhering sand to the maize root which was gained by shaking the root), and rhizosphere soil II (rII, remaining sand which was washed from the root). Df is degrees of freedom, values are F-statistics derived from permutation and ANOVA tests. ^∗^p < 0.05, ^∗∗^p < 0.01, ^∗∗∗^p < 0.001.*

### Metabolome

**FIGURE 3:**
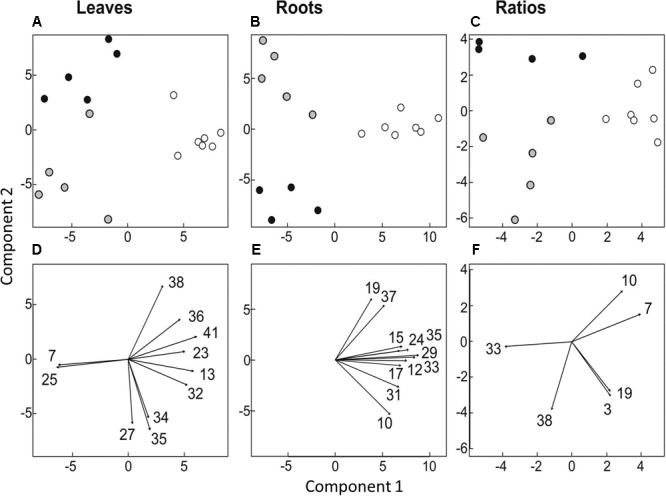
Multivariate ordination analysis [partial least square-discriminant analysis (PLS-DA)] of metabolite levels in leaves **(A,D)** and roots **(B,E)** and of leaf/root ratios **(C,F)**. **(A–C)** Separation of treatments: Plants not treated with protists are represented by white circles (*n* = 10), plants treated with protists once by gray circles (*n* = 5) and plants treated twice with protists by black circles (*n* = 5). **(D–F)** Metabolite levels or ratios correlating significantly with PLS-DA data (*p* < 0.01) are indicated by arrows. The numbers given refer to respective numbers in **Table 2**.

**FIGURE 4:**
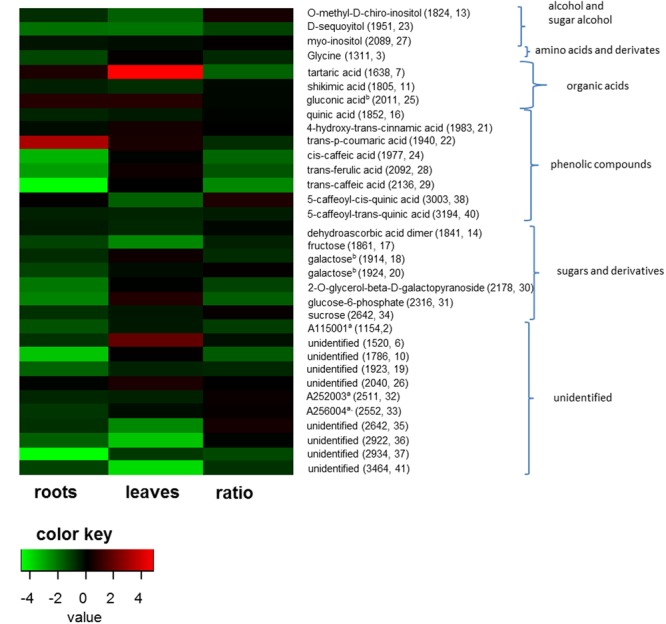
Fold changes of metabolite levels in roots, leaves and ratios from plants inoculated with protists (*n* = 10) compared to not inoculated plants (*n* = 10). Fold changes were logarithmized for better visualization. Only fold changes lower than 0.66 and higher 1.5 are shown. Color key represents fold changes of the metabolites from strong decrease (green) to high increase (red) in the presence of protists. Ratio shows the fold changes of leaf/root ratios. Parentheses show the retention time and internal numbering according to **Table 2** of the metabolites.

Differences in organ-specific metabolite levels and in metabolite ratios (leaf/root) induced by the presence of protists were analyzed by PLS-DA. Metabolite profiles of plants from the different treatments could be separated in all cases from each other (**Figures 3A–C**). Plants with and without added protists were separated along the first component and plants differing in the number of protist additions were separated along the second component. This separation was highly significant for root metabolites (PERMANOVA, *p* = 0.001) and significant for leaf metabolites (PERMANOVA, *p* = 0.012), but not significant for metabolite ratios. Nevertheless, several specific metabolite ratios, such as that of glycine, tartaric acid, 5-caffeoyl-*cis*-quinic acid significantly correlated with the separation of the treatments (**Figure 3F** and **Table 2**). Differences on the level of the individual organs (roots or leaves) were more pronounced when compared to differences in the leaf/root-ratios (**Figures 3D–F**). Most of the significant changes and correlations corresponded to decreased levels of certain metabolites in the plants inoculated with protists, in particular in the roots (**Figure 4** and **Table 2**). According to PLS-DA this applies to O-methyl-D-chiro-inositol, D-sequoyitol, myo-inositol, sucrose, and 5-caffeoyl-*cis*-quinic acid in leaves and to citric acid, *cis*- and *trans*-caffeic acid and glucose-6-phosphate in roots. The student *t*-test (two-sided *t*-test) identified seven compounds from leaves (xylose, shikimic acid, O-methyl-D-chiro-inositol, dehydroascorbic acid, fructose, D-sequoyitol, 5-caffeoyl-*trans*-quinic acid, 16 degrees of freedom) and 11 compounds from roots (xylitol, shikimic acid, citric acid, dehydroascorbic acid, quinic acid, fructose, galactose, *cis*-caffeic acid, 2-O-glycerol-beta-D-*trans*-caffeic acid, glucose-6-phosphate, sucrose, 15 degrees of freedom) with significantly reduced levels in plants inoculated with protists (**Table 2**). In contrast, we observed an upregulation of metabolite levels for pyroglutamic acid, tartaric acid, galactose and 4-hydroxy-*trans*-cinnamic acid, gluconic acid, *trans*-ferulic acid and glucose-6-phosphate in leaves and for malic acid and *trans*-*p*-coumaric acid in roots upon inoculation with protists (**Figure 4** and **Table 2**). Aside from pyroglutamic acid we could not observe any significant increase of N and P containing metabolites (**Table 3**) in the maize plants grown in the presence of protists.

**Table 2:** Significant fold changes of metabolite levels analyzed with GC-MS or ratios from maize plants inoculated with protists compared to plants not inoculated with protists.

Figure 3	Cluster time	Compound	Fold change leaf	Fold change root	Fold change ratio
1	1029	Unidentified	**0.79**	0.97	0.95
2	1154	A115001^a^	**0.70**	0.24	0.37
3	1311	Glycine	1.21	0.30	*0.52*
4	1486	Malic acid	1.13	**1.46**	0.94
5	1517	Pyroglutamic acid	**1.38**	1.11	1.03
6	1520	Unidentified	**6.89**	0.43	0.83
7	1638	Tartaric acid	***125.02***	1.90	***0.18***
8	1657	Xylose^b^	**0.76**	0.73	0.98
9	1719	Xylitol^b^	0.96	**0.66**	1.12
10	1786	Unidentified	1.20	***0.03***	***0.21***
11	1805	Shikimic acid	**0.49**	**0.63**	0.93
12	1815	Citric acid	1.06	***0.72***	1.10
13	1824	O-methyl-D-chiro-inositol	***0.19***	0.47	1.70
14	1841	Dehydroascorbic acid dimer	**0.52**	**0.69**	0.99
15	1846	Unidentified	**0.69**	***0.69***	1.00
16	1852	Quinic acid	0.69	**0.56**	1.01
17	1861	Fructose	**0.09**	***0.32***	0.62
18	1914	Galactose^b^	**1.47**	0.51	0.51
19	1923	Unidentified	0.58	*0.18*	*0.54*
20	1924	Galactose^b^	0.88	**0.32**	**1.21**
21	1938	4-hydroxy-*trans*-cinnamic acid	**1.64**	0.89	**1.16**
22	1940	*Trans*-*p*-coumaric acid	1.76	**25.82**	0.49
23	1951	D-sequoyitol	***0.13***	0.14	0.32
24	1977	*Cis*-caffeic acid	1.03	***0.04***	**0.17**
25	2011	Gluconic acid^b^	***2.25***	2.18	1.00
26	2040	Unidentified	**1.87**	1.01	**1.15**
27	2089	Myo-inositol	*0.86*	0.75	1.24
28	2092	*Trans*-ferulic acid	**1.47**	0.06	0.23
29	2136	*Trans*-caffeic acid	1.16	***0.01***	**0.09**
30	2178	2-O-glycerol-beta-D-galactopyranoside	1.05	**0.12**	0.33
31	2316	Glucose-6-phosphate	**2.09**	***0.10***	**0.20**
32	2511	A252003^a^	***0.62***	0.54	1.37
33	2552	A256004^a^	0.88	***0.42***	***1.29***
34	2642	Sucrose	*0.72*	**0.46**	1.26
35	2642	Unidentified	*0.09*	0.45	1.65
36	2922	Unidentified	***0.03***	0.19	1.01
37	2934	Unidentified	0.39	***0.01***	**0.29**
38	3003	5-caffeoyl-*cis*-quinic acid	*0.19*	1.19	*2.00*
39	3008	Unidentified	**0.69**	1.19	1.04
40	3194	5-caffeoyl-*trans*-quinic acid	**0.56**	0.60	0.65
41	3464	Unidentified	***0.02***	0.33	0.44

*Significant fold changes according to a student t-test (p < 0.05) are marked by bold letters; a significant correlation of respective metabolites or metabolite ratios with the separation of treatments in partial least square-discriminant analysis (PLS-DA) (p < 0.01) is marked by italic letters. ^*a*^From the Golm metabolome database. ^*b*^Retention time index and fragmentation pattern were not sufficient to differentiate between closely related isomers*.

**Table 3:** Fold changes of P and N containing metabolites analyzed with GC-MS or ratios from maize plants inoculated with protists compared to plants not inoculated with protists. Metabolites containing P are listed above, metabolites containing N below the dashed line.

Cluster time	Compound	Fold change leaf	Fold change root	Fold change ratio
1274	Phosphoric acid	1.15	0.85	1.06
1311	Glycine	1.21	0.30	*0.52*
1428	Aspartic acid	1.16	1.16	0.99
1517	Pyroglutamic acid	**1.38**	1.11	1.03
1530	Glutamic acid	1.23	0.23	0.32

*Significant fold changes according to a student t-test (p < 0.05) are marked by bold letters; a significant correlation of respective metabolites or metabolite ratios with the separation of treatments in partial least square-discriminant analysis (PLS-DA) (p < 0.01) is marked by italic letters.*

## Discussion

A positive impact of the presence of rhizosphere protists on plant productivity has been described repeatedly. Studies with *Plantago lanceolata L.* using growth periods of >30 days could detect a clear increase in root and shoot biomass in the presence of protists (Koller et al., 2013a,b). Inoculation of plants with amoebae resulted in changes in the root architecture of rice (*Oryza sativa* L.), i.e., the root system became more ramified thus increasing the nutrient uptake efficiency of the plant (Kreuzer et al., 2006). Similarly, studies with *Arabidopsis thaliana* found an increase in rosette diameter and shoot biomass in the presence of *Acanthamoeba castellanii* after 6 days of incubation (Krome et al., 2009). Additional evidence has been presented for an impact of protists on microbial plant hormone production (Bonkowski and Brandt, 2002) due to grazing-induced proliferation of bacteria producing auxin. The combined effect of hormonal feed-back (Bonkowski, 2004) and increased nutrient availability (Koller et al., 2013b) is likely resulting in increased investment of the plant into the root system as a hot-spot for bacteria-protists interactions (Jousset, 2017). The definitive explanatory mechanisms responsible for these effects, however, have not been determined conclusively and require additional experimental approaches in the future. We monitored protist-induced shifts in above-and below-ground maize plant metabolite profiles to more closely delimit possible bottom-up mechanisms for the impact of rhizosphere protists on the plants. So far there exist some examples for changes in gene expression after incubation with PGPR for maize (Li et al., 2014), Arabidopsis (Sukweenadhi et al., 2015), and oil palm (Lim et al., 2010), mostly in response to stress and not with regard to rhizosphere interactions.

In accordance with Rosenberg et al. (2009), who described selective grazing by protists in the rhizosphere, protists had a clear effect on the overall bacterial community composition, richness and evenness in our microcosms. Bacterial communities exposed to protists did not vary as much as bacterial communities thriving without protists (**Figure 2**). Rosenberg et al. (2009) had shown that *Betaproteobacteria* and *Firmicutes* were reduced in the presence of *A. castellanii* and Koller et al. (2013b) detected a particular decrease in gram-negative bacteria using the same amoeba. Since the top-down control of prey communities depends amongst others on predator identity (Saleem et al., 2012, 2013) our experiment included a mixture of protists to account for diverse feeding strategies, which would be closer to natural conditions. *Tetrahymena pyriformis* is a filter-feeding ciliate, which is very effective in taking up small, suspended particles via a current created by its cilia (Fenchel, 1987; Parry, 2004). The heterotrophic flagellate *Cercomonas longicauda* possesses two flagellas and produces filose pseudopods to capture bacteria selectively (Bass et al., 2009; Pedersen et al., 2008). *Acanthamoeba polyphaga* shows raptorial-feeding and amoeba are supposed to be the dominant bacterial consumers in soil (Clarholm, 1981; Weekers et al., 1993). While a deeper analysis of the community composition using sequence-based approaches was beyond the scope of this work, our results clearly show that T-RFLP is sufficient to reveal the shifts in community composition as a response to protist grazing. Comparisons between sequence-based approaches and T-RFLP profiling have shown repeatedly that, e.g., correlation of the microbial communities with environmental factors is consistent with both approaches (Pilloni et al., 2012; de la Fuente et al., 2014). T-RFLP is thus a reliable tool to rapidly observe shifts in communities over time or distances (Giebler et al., 2013; Glaser et al., 2014, 2015), even though the choice of different restriction enzymes may result in variable species richness and diversity indices (Zhang et al., 2008) Differences between the three soil fractions (bulk soil, rhizosphere soil, rhizosphere II) were not detectable, which could be due to the experimental setup. As sand has a high pore capacity, the demarcation of bulk soil and rhizosphere soil may not have been as prominent as in natural soil. In contrast to the clear effects of protist grazing on bacterial communities, we observed only slight significant effects of protist treatments on the measured plant growth parameters. This poor responsiveness may be due to our chosen model plant or to the relatively short experimental time of 14 days.

While metabolite profiles may be relatively variable and subject to multiple external factors, they provide a holistic and sensitive image of the state of a plant organism (Fester et al., 2014). This sensitivity allowed the detection of a considerable number of clear and significant changes in the levels of individual metabolites, despite of the lack of clear effects on plant growth parameters. There was a surprisingly large amount of changes in the levels of polyols (xylitol, O-methyl-D-chiro-inositol, D-sequoyitol, myo-inositol), and of a number of carbohydrates (xylose, fructose, galactose, 2-O-glycerol-beta-D-galactopyranoside) which are typically upregulated in plant stress response and which are discussed to have antioxidant capacities (Sardans et al., 2011; Jorge et al., 2016; Goufo et al., 2017). Changes in the levels of dehydroascorbic acid which are part of the antioxidant metabolism can be interpreted in a similar way (Pereira et al., 2014). In addition there were many changes in metabolites connected to the metabolism of phenolic acid (Caretto et al., 2015) such as shikimic acid, quinic acid, 4-hydroxy-*trans*-cinnamic acid, *trans*-*p*-coumaric acid, *cis*-caffeic acid, *trans*-ferulic acid, *trans*-caffeic acid, 5-caffeoyl-*cis*- and *trans*-quinic acid. Such compounds are important in plant defense and are often produced under stress conditions (Caretto et al., 2015).

Since most of the stress-related metabolites were observed in lower levels in plants inoculated with protists, our measurements provide evidence for a reduction of plant stress levels in the presence of rhizosphere protists and their selective influence on bacterial community composition. Apart from an impact on plant stress levels, protist also may improve plant mineral nutrition (Kuikman and Van Veen, 1989; Koller et al., 2013b). In prior metabolite profiling experiments, improvements in mineral nutrition were consistently reflected by increased levels of amino acids like glutamic or aspartic acid (Fester et al., 2011, 2014). We did not observe an increase in these metabolites in the current experiment, nor did we observe similarly indicative shifts (Fester et al., 2013) in root/shoot ratios of these metabolites (**Table 3**). Our data, thus do not allow to completely exclude a nutritive effect of protists in our experiment. Overall, the downregulation of stress related metabolites in the presence of protists could be a hint for so far unknown hidden bottom-up effects of protist-bacteria interactions on the plant metabolic state in addition to the already known nutritive effects. A so far undescribed direct protist-plant interaction might also explain the plant response; distinguishing the direct or indirect effect of rhizosphere protists requires, however, additional future studies.

Contrary to our observation of reduced stress-related metabolites, other studies showed an increase in proteins related to stress together with an upregulation of metabolites for photosynthesis, hormone biosynthesis and tricarboxylic acid cycle in the presence of PGPR in maize plants (Li et al., 2014). However, Li et al. (2014) had included stress as an experimental factor in their experiment. Transcript analysis of oil palm roots incubated with PGPR also showed an upregulation of genes involved in stress in addition to protein synthesis, primary metabolism and membrane transport (Lim et al., 2010). The incubation of wheat with PGPR showed an enhancement in the expression of genes related to nutrient acquisition, nitrogen assimilation, DNA replication and regulation of cell division (Camilios-Neto et al., 2014). These examples demonstrate that, while the effects of PGPR on plants are diverse and apparently depend on the composition of respective bacterial communities, root-associated bacterial communities can modify plant stress levels. As the most likely explanation for the observed concomitant shifts in microbial communities and plant stress levels we therefore assume that protist actions increased the abundance of microorganisms with positive effects for maize plants and decreased the abundance of microorganisms with negative effects. Such strong effects of predators on bacterial community composition and function have already been shown (Rønn et al., 2002; Blanc et al., 2006).

Since resource allocation for plant defense may compete with resource allocation for plant growth (Herms and Mattson, 1992), a decrease in plant stress levels caused by the presence of rhizosphere protists may well be an explanation for correlating growth effects described in other experiments (Krome et al., 2009; Koller et al., 2013a,b). In summary, our results indicate that a decrease in plant stress levels, most likely caused by protist-induced shifts in microbial communities, is a prominent effect of microbial predator-prey interactions in the rhizosphere. Depending on conditions, this effect may well affect plant growth and should therefore be included as a possible mechanism when studying the impact of rhizosphere protists on plants. It should also be taken into account that sensitive indicators like metabolites are necessary to capture all effects of rhizosphere interactions.

## Author Contributions

AK and AC conceived and designed the research. AK conducted the experiments. AK, TF, and CH analyzed the data. All authors wrote, read, and approved the manuscript.

## Conflict of Interest Statement

The authors declare that the research was conducted in the absence of any commercial or financial relationships that could be construed as a potential conflict of interest.
